# Interconnected Clinical and Social Risk Factors in Breast Cancer and Heart Failure

**DOI:** 10.3389/fcvm.2022.847975

**Published:** 2022-05-20

**Authors:** Arjun Sinha, Avni Bavishi, Elizabeth A. Hibler, Eric H. Yang, Susmita Parashar, Tochukwu Okwuosa, Jeanne M. DeCara, Sherry-Ann Brown, Avirup Guha, Diego Sadler, Sadiya S. Khan, Sanjiv J. Shah, Clyde W. Yancy, Nausheen Akhter

**Affiliations:** ^1^Division of Cardiology, Department of Medicine, Northwestern University Feinberg School of Medicine, Chicago, IL, United States; ^2^Department of Medicine, Northwestern University Feinberg School of Medicine, Chicago, IL, United States; ^3^Department of Preventive Medicine, Division of Cancer Epidemiology and Prevention, Northwestern University Feinberg School of Medicine, Chicago, IL, United States; ^4^UCLA Cardio-Oncology Program, Division of Cardiology, Department of Medicine, University of California, Los Angeles, Los Angeles, CA, United States; ^5^Division of Cardiology, Department of Medicine, Emory University, Atlanta, GA, United States; ^6^Division of Cardiology, Department of Medicine, Rush University Medical Center, Chicago, IL, United States; ^7^Section of Cardiology, Department of Medicine, University of Chicago Medicine, Chicago, IL, United States; ^8^Division of Cardiology, Department of Medicine, Medical College of Wisconsin, Milwaukee, WI, United States; ^9^Cardio-Oncology Program, Division of Cardiology, Medical College of Georgia at Augusta University, Augusta, GA, United States; ^10^Cardio-Oncology Program, Division of Cardiology, The Ohio State University Medical Center, Columbus, OH, United States; ^11^Robert and Suzanne Tomsich Department of Cardiovascular Medicine, Heart, Vascular and Thoracic Institute, Cleveland Clinic Florida, Weston, FL, United States

**Keywords:** breast cancer, heart failure, risk factors, social determinants of health, reverse cardio-oncology

## Abstract

Breast cancer and heart failure share several known clinical cardiovascular risk factors, including age, obesity, glucose dysregulation, cholesterol dysregulation, hypertension, atrial fibrillation and inflammation. However, to fully comprehend the complex interplay between risk of breast cancer and heart failure, factors attributed to both biological and social determinants of health must be explored in risk-assessment. There are several social factors that impede implementation of prevention strategies and treatment for breast cancer and heart failure prevention, including socioeconomic status, neighborhood disadvantage, food insecurity, access to healthcare, and social isolation. A comprehensive approach to prevention of both breast cancer and heart failure must include assessment for both traditional clinical risk factors and social determinants of health in patients to address root causes of lifestyle and modifiable risk factors. In this review, we examine clinical and social determinants of health in breast cancer and heart failure that are necessary to consider in the design and implementation of effective prevention strategies that altogether reduce the risk of both chronic diseases

## Introduction

Cardiovascular disease (CVD) and cancer are the two leading causes of death in the United States in 2020 (1). Classically, the field of cardio-oncology has focused on the development of CVD directly from cardiotoxic effects of cancer biology and/or cancer therapies. But there is growing appreciation that the two diseases intersect at multiple levels, including shared clinical risk factors, shared social risk factors, and reverse cardio-oncology where CVD acts to promote cancer development (2). In this review, we focus specifically on the intersections between breast cancer and heart failure (HF). Delving into anti-cancer therapies that cause cancer therapy-related cardiac dysfunction is beyond the scope of this review. Breast cancer remains the most common cancer in women, with one in eight women expected to develop breast cancer over the course of their lifetime (3). There have been notable improvements in survival rates for breast cancer due to earlier detection and advancements in treatment such that the 5-year relative survival rate from the mid-1970s to the present time has increased from 75 to 90% (4). Breast cancer survivors with a prior history of CVD who survive cancer for over 5 years are more likely to die of CVD, (5) and in breast cancer survivors age 66 years or older, CVD is often the primary cause of death (6). The lifetime risk of developing HF in women is even higher at one in five at age 40 and rises rapidly with increasing age (7, 8). The overall burden of HF continues to increase with the aging of the general population and with increases in HF risk factors such as obesity and diabetes. Thus, as women age they are at increased risk for both breast cancer and HF. Here we examine the shared pathophysiology and commonalities in clinical and social risk factors that lead to the high prevalence of both HF and breast cancer.

### Shared Clinical Risk Factors

Traditional clinical risk factors for HF in women are well established. Modifiable clinical HF risk factors that may also increase risk for breast cancer include diabetes, obesity, hypertension, hyperlipidemia, and atrial fibrillation (Table 1) (9). Inextricably linked with these risk factors are health behaviors such as tobacco use, alcohol use, physical inactivity, and an unhealthy diet. Prevention of risk factors (or primary prevention) and avoidance of poor health behaviors dramatically lower the risk of incident HF (10, 11). The causal pathways connecting these risk factors to increased risk of CVD and HF are well known (12–14). However, their association with increased risk of breast cancer is only starting to be appreciated (15). In this section, we summarize epidemiological and mechanistic evidence to better understand the relationship between some of the traditional cardiovascular risk factors and breast cancer.

**Table 1:** Impact of modifiable heart failure risk factors that increase risk for breast cancer and potential underlying mechanisms.

**Modifiable heart failure risk factors**	**Risk of breast cancer**	**Mechanisms**
Diabetes	20% Increased Risk (19)	Hyperinsulinemia. Adipocyte Dysfunction. Hypoxia. Immune Cell Recruitment. Expression of Aromatase. Hyperleptinemia
Obesity	25% Increased Risk (20)	
Hypertension	15% Increased Risk (36)	Angiotensin II
Hyperlipidemia	9% Increased Risk (41)	27-hydroxycholesterol
Atrial Fibrillation	35% Increased Risk (47)	Reactive Oxygen Species

#### Obesity and Glucose Dysregulation

Of the common modifiable risk factors, diabetes and obesity have the strongest association with HF in women (16, 17). Multiple studies have also shown an increased risk of breast cancer in women with diabetes. In a meta-analysis of 39 independent risk estimates from observational epidemiological studies, women with diabetes had a 27% higher risk of developing breast cancer (summary relative risk [SRR] 1.27, 95% confidence interval [CI], 1.16 – 1.39) (18). In prospective studies, the risk of developing breast cancer remained 23% higher in women with diabetes (SRR 1.23 [95% CI, 1.12–1.35]). Part of the risk was mediated through concomitant obesity, but the risk of developing breast cancer remained 16% higher after adjusting for body mass index (BMI). Of note, the risk of breast cancer was not elevated in premenopausal women with diabetes or women with Type 1 diabetes. A more recent review of meta-analyses estimated a 20% greater risk of developing breast cancer in women with diabetes (19). Similarly, the risk of breast cancer is 25% higher in post-menopausal women with obesity (20). The risk of breast cancer increases by 10% for every 5 kg/m^2^ higher BMI above 25 kg/m^2^ in postmenopausal women (21). This association is strongest in estrogen receptor positive breast cancer (22). Obesity contributes to a chronic low-grade inflammation that can promote both carcinogenesis and atherosclerosis. Changes in the adipose tissue microenvironment can switch from anti-inflammatory to pro-inflammatory in obesity (23).

Glucose dysregulation is central to both disease processes and is integral to understanding the pathophysiology underlying this association. Both obesity and diabetes lead to adipocyte dysfunction, insulin resistance, and hyperglycemia (24, 25). The excess growth of adipose tissue results in hypoxia and expression of hypoxia-inducible factor 1a (HIF1a) (26). This results in adipocyte dysfunction, which promotes breast cancer growth through multiple interconnected pathways. First, adipocyte hypoxia results in release of chemokines such as monocyte chemoattractant protein 1 (MCP1), which recruits immune cells and creates a pro-inflammatory environment (27). Second, there is increased expression of aromatase, the rate-limiting enzyme in estrogen synthesis, which leads to higher levels of circulating estrogen (28). Higher levels of estrogen promote estrogen-responsive malignancies including breast cancer. Third, there is dysregulation of adipocyte endocrine function. In individuals with obesity, the central nervous system develops resistance to leptin, a hormone that limits appetite in healthy individuals (29). The subsequent hyperleptinemia promotes breast cancer initiation, growth, and progression by promoting cellular growth, inhibiting apoptosis, activating cellular adhesion and inflammatory immune cells (30). In contrast, in obesity there is reduced production of protective hormones such as adiponectin and ghrelin, both of which reduce breast cancer risk by inhibiting aromatase and other pathways associated with increased cancer cell proliferation (31, 32).

In combination with inflammatory cytokines, hypoxia, elevated estrogen, and altered milieu of adipokines, hyperinsulinemia and hyperglycemia lead to dysregulation of multiple metabolic pathways in not only breast cancer cells but also local stromal and immune cells (33). These triggers stimulate signaling cascades by activating receptor tyrosine kinases leading to activation of the phosphoinositide 3-kinase (PI3K)-AKT pathway and inhibition of the AMP-activated protein kinase (AMPK); favoring a shift toward aerobic glycolysis, glucose uptake, and cell proliferation in cancer, stromal, and immune cells (34, 35). These pathways also lead to aromatase activation in stromal cells and release of inflammatory cytokines from immune cells resulting in a positive feedback cycle and tumor progression (33).

#### Hypertension

Hypertension is one of the most prevalent risk factors for both HF and breast cancer, especially as the population ages. Numerous observational studies have also evaluated the association of hypertension with risk of incident breast cancer. A large meta-analysis of 30 observational studies, including 11,643 cases of breast cancer, demonstrated a 15% higher risk of breast cancer in adults with hypertension (RR: 1.15; 95% CI 1.08 – 1.22) (36). In another meta-analysis of 13 prospective studies, the association between hypertension and breast cancer was again noted (RR: 1.07; 95% CI 0.84 – 1.35) (37). This was primarily driven by the association observed in postmenopausal women. Like diabetes, hypertension was not associated with increased risk of breast cancer among premenopausal women. Mechanisms behind hypertension and breast cancer risk are not well established. Since hypertension is often linked with diabetes and obesity, there are some shared pathways such as chronic inflammation as described above. One specific pathway that links both obesity and hypertension to breast cancer involves angiotensin II. While the renin-angiotensin system is well-known for its role in blood pressure and fluid regulation, it can be activated within dysregulated adipose tissue as well (38). Angiotensin II increases tumor angiogenesis in receptor-negative breast cancer and leads to activation of proinflammatory macrophages promoting tumor growth.

#### Cholesterol Dysregulation

Dysregulation in cholesterol metabolism is another traditional cardiovascular risk factor that is associated with breast cancer. Some studies have demonstrated an association between high-density lipoprotein cholesterol (HDL-C) and breast cancer risk (39). In a study of 4,670 women with increased mammographic density, higher levels of HDL-C were associated with a 23% increased risk of breast cancer (40). While observational data have not consistently shown an association between low-density lipoprotein cholesterol (LDL-C) and breast cancer risk, a large mendelian randomization of > 400,000 participants found a significant association between genetic risk factors for lifelong elevated LDL-C and increased risk of estrogen receptor positive breast cancer (41). There is also evidence that higher dietary intake of cholesterol is associated with an increased risk of breast cancer in a non-linear fashion (42). However, it is difficult to disentangle the effects of obesity and diabetes from hypercholesterolemia using observational data.

There is growing mechanistic evidence that links hypercholesterolemia with breast cancer. 27-hydroxycholesterol is an endogenous oxysterol that has activity as a selective estrogen receptor modulator (43). It is generated by the P450 enzyme sterol 27-hydroxylase CYP27A1 and is transported in conjunction with HDL-C and LDL-C. It has been shown to stimulate the growth of estrogen receptor positive breast cancer cells in human xenografts and animal models. Potential mechanisms include inhibition of tumor suppressor proteins, activation of growth factors, and immune dysregulation such as suppression of cytotoxic CD8+ T cells within tumors (44). More work is needed to better understand this pathway and how cholesterol lowering therapies such as statins may affect it. Current data do not show convincing evidence of statin therapy protecting against breast cancer development but there are multiple observational studies suggesting a benefit of lipophilic statins on breast cancer recurrence and mortality (45).

#### Atrial Fibrillation

There is an association between atrial fibrillation (AF) and cancer, with inflammation contributing to the development of both in part through the production of reactive oxygen species. Elevation in C-reactive protein levels and increased NLRP3 inflammasome activation have also been reported in AF (46). Whether atrial fibrillation itself increases the risk of developing cancer requires further investigation. In a cohort study of 34,691 women followed for a median of 19 years, new-onset AF was found to be a significant risk factor for incident breast cancer after age-adjusted models (hazard ratio [HR], 1.35; 95% CI, 1.01–1.81; *p* < 0.04). This risk was highest in the first 3 months after incident AF, but remained beyond 1 year (47). Atrial fibrillation may also be a marker for occult cancer. Patients with cancer have a higher prevalence of AF compared to those in the general population (48). Women with breast cancer diagnosis have a significantly higher incidence of AF, with increasing risk for those who present at a higher breast cancer stage. Incident AF in newly diagnosed breast cancer also increases 1-year CV mortality (49).

#### Inflammation

As described above, immune dysregulation and inflammation are common final pathways that link traditional HF risk factors to breast cancer development. Obesity can lead to a chronic low-grade inflammation which leads to accumulation of pro-inflammatory adipose tissue macrophages, increased levels of aromatase, estrogen biosynthesis, and increased risk for estrogen-dependent breast cancer after menopause (28). Some inflammatory pathways are shared in HF and cancer pathogenesis. Pro-inflammatory cytokines such as tumor necrosis factor (TNF), interleukin (IL)-1B, IL-6, and IL-18 have been shown to play a role in left ventricular dysfunction and adverse remodeling (50, 51). Increased expression of these cytokines, especially IL-1B, is due to activation of the NLRP3 inflammasome (52). The Canakinumab Anti-Inflammatory Thrombosis Outcome Study (CANTOS) evaluated the effect of canakinumab, a monoclonal antibody targeting interleukin-1B (IL-1B), on cardiovascular outcomes (53, 54). Canakinumab significantly reduced not only cardiovascular events and HF hospitalizations but incident lung cancer and decreased lung cancer-related death. While the trial did not have enough power to look at different cancer subtypes, breast cancer tumor cells have been shown to produce IL-1B, which promotes epithelial-to-mesenchymal transition, migration, and invasion of breast cancer cells (55). Animal models have shown reduction in breast cancer metastasis with IL-1B inhibition (55). Identification of these shared pathways may allow for targeted therapies for both breast cancer and HF.

### Shared Social Risk Factors

Poverty and inequality form the backbone of underlying social risk factors that contribute to social determinants of health (SDOH). These are primary concerns for healthcare providers who must consider community-level factors that influence health outcomes. Thriving in a society involves addressing a complex association between personal, environmental, economic, and social factors that impact overall health. There are multiple SDOH assessment tools which have been developed to comprehensively evaluate these outcomes. SDOH screening tools must be better integrated into healthcare delivery schema in cardio-oncology. Several social risk factors derived from these tools are known to contribute to both cancer and HF, including socioeconomic status, neighborhood disadvantage, food insecurity, an inadequate healthcare system (lack of insurance, cost of medication), and social isolation (56, 57). These social issues have come to the forefront during the COVID-19 pandemic, where we have witnessed the selective effect of COVID-19 on disadvantaged communities. The pandemic has motivated a conversation to address these disparities in healthcare, which are deeply rooted in the structural inequities in our society. Potential mitigation strategies must be directed at multiple levels (Table 2). In this section, we summarize social risk factors that contribute to both breast cancer and HF.

**Table 2:** Common social risk factors between heart failure and breast cancer.

**Social risk factor**	**Heart failure**	**Breast cancer**	**Potential solutions**
Low socioeconomic status	↑ Incidence of disease ↑ Mortality after 90 days of discharge ↓ Likely to be referred to subspecialist ↑ Hospitalizations, readmissions, and mortality	↑ Incidence of disease ↑ Aggressive premenopausal breast cancer ↑ Stage of breast cancer diagnosis ↑ Mortality	- Create a robust income safety net - Increase income benefits - Increase jobs/employment - Expand unemployment insurance
Neighborhood disadvantage	↑ Incidence of disease ↓ Ejection fractions ↑ Hospitalizations, readmissions, and mortality	↑ Stage of breast cancer diagnosis ↑ Breast cancer mortality	- Create a robust income safety net - Increase affordable public housing; prioritize for homeless - Rental assistance - Investment in low-income communities - Investment in schools, early childhood education, and mentorship programs - Build affordable transportation - Partner with social services addressing homelessness
Food insecurity	- Frailty and deconditioning - Poor access to low-sodium diet - Obesity, diabetes, hypertension more prevalent	- Dietary fat linked to reduced breast cancer - Obesity, diabetes, hypertension more prevalent	- Create a robust income safety net - Address food deserts - Expand food benefits - Expand universal free meals to children - Partner with local food banks and fridges
Poor access to healthcare	- Lack of continuity care - Lack of subspeciality care ↑ Medication costs	↓ Cancer screening ↑ Delays in diagnosis and treatment of breast cancer	- Create a robust income safety net - Affordable healthcare - Universal healthcare - Partner with community health centers - Prioritize access in health services
Social isolation	↓ Physical and mental health ↑ Hospitalizations, readmissions and mortality	↓ Physical and mental health ↓ Survival	- Access to mental health services - Increase social workers on healthcare teams - Patient support groups - Partner with local programs for the elderly

#### Socioeconomic Status

There is a known association between socioeconomic characteristics and risk for both breast cancer and HF. Across racial and ethnic groups, increasing socioeconomic status is inversely correlated with breast cancer incidence in population studies (58). Low socioeconomic status is associated with increased risk of aggressive premenopausal breast cancer, later stage of diagnosis, and poorer survival (56). Breast cancer's 3-year survival is significantly affected by level of education, district of residence and social class in childhood (59). Mortality is significantly higher in non-Hispanic Black breast cancer patients than non-Hispanic white patients, across all ages (60). Cardiovascular health is also worse in Black individuals who have a higher prevalence of HF risk factors such as obesity, diabetes, and hypertension than non-Hispanic white individuals (61, 62). Black individuals have higher rates of HF hospitalization and age-adjusted HF-related CVD death rates than their White counterparts (63). When compared to White survivors of breast cancer, Black survivors have an elevated risk of cardiotoxicity-associated morbidity and mortality (64).

Socioeconomic factors also predict outcomes from HF admissions. Those patients with adverse social factors in a Medicare dataset were 3-fold as likely to die within 90 days of discharge for a HF hospitalization as those without any social risk factors (65). In a study from Sweden evaluating HF outcomes, lower socioeconomic status was directly associated with patients being less likely to have a subspecialist referral (66). This may in part be due to the financial burden of care for cancer patients which is even higher when superimposed with atherosclerotic CVD (a major risk factor for HF), leading to difficulty paying bills, buying medications, and seeking care (67).

There is a linear relationship between number of socioeconomic risk factors and higher risk of HF hospitalization, cardiovascular events, and mortality (66). Prevention focusing on modifiable clinical risk factors is difficult for patients without socioeconomic support and resources. A patient with income instability must prioritize housing, food, utilities, and other needs over healthy activities such as a moderate-intensity exercise routine. Lower socioeconomic status is associated with a significant increase in body mass index, smoking prevalence, and diabetes (68). Other major risk factors for HF including coronary artery disease and hypertension also vary widely with levels of adverse social factors (69). Due to these underlying risk factors, those patients from lower socio-economic classes have a higher prevalence of incident HF 5 years earlier than those from more affluent backgrounds (68).

#### Neighborhood Disadvantage

Poor infrastructure and inadequate resources in low-income neighborhoods serve as barriers to healthcare. Housing insecurity, the role of public transportation, and travel costs may serve as physical impediments to access healthcare but have not been well studied. Geographic proximity and travel time to mammography facilities have not been shown to be associated with later stage breast cancer diagnosis (70). However, high census tract poverty (defined by the US census as > 20% below poverty) and inner-city disadvantage have shown an association with risk of later stage breast cancer diagnosis (70, 71). In addition, concern for safety due to neighborhood violence or crime, lack of public spaces such as parks, and lack of exercise facilities can lead to a less active and more sedentary lifestyle. Obesity is highly correlated with neighborhood poverty (71), having both direct and indirect effects on breast cancer and HF. Stressors associated with poverty can cause a patient to turn to risky behaviors such as smoking, drinking, and drug use as coping mechanisms. High-income neighborhoods have demonstrated lower stress, anxiety, rates of obesity, and fewer other comorbidities (72).

Neighborhood deprivation index includes four main components: wealth and income, education, occupation, and housing quality (73). Akwo and colleagues demonstrated that neighborhood deprivation predicts risk of incident HF beyond individual socioeconomic status and traditional cardiovascular risk factors in low-income populations (74). Residents living in deprived neighborhoods have lower ejection fractions, more severe HF symptoms and higher odds of hospitalization for HF (75). Thirty-day HF readmission and mortality rates also increase with neighborhood deprivation (76). Neighborhood socioeconomic status is also an important factor in cancer-specific survival disparities in Black and non-Hispanic Whites (77).

#### Food Insecurity

Food insecurity is the lack of reliable access to nutritious food for healthy and active living, resulting in not having enough meals or cutting back on meals. It is a broad concept of adapting eating to social circumstances primarily driven by poverty, income instability, and neighborhood disadvantage. Food deserts are areas in primarily low-income neighborhoods where access to grocery stores that provide fresh fruits and vegetables is limited (78). This may also contribute to difficulty in adhering to a low-sodium diet for patients with HF when facing food insecurity. For patients with breast cancer and HF, food insecurity can have the potential to aggravate both conditions. Food insecurity and lack of healthy food is associated with HF risk factors and HF, but whether food insecurity and access to healthy food is associated with breast cancer requires further study.

One hypothesized mechanism for the association of SDOH and risk of HF is lack of access to healthy foods, more processed foods, and therefore higher dietary phosphate intake, which may increase circulating levels of inorganic phosphate and fibroblast growth factor 23 (FGF-23). FGF-23 has been correlated with increased myocardial fibrosis on cardiac MRI and a strong predictor of mortality and first HF hospitalization, especially in patients with HF with preserved ejection fraction (79). Further investigation is needed to understand the relationship between a high phosphate diet and breast cancer. Ultra-processed foods in diet have been associated with increased risks of overall and breast cancer (80). There may also be a possible link between lipids, higher HDL-C and apolipoprotein A1, and mammographic density which needs further study (40). A few studies have noted that dietary fat, n-3 PUFA, has an inverse link to breast cancer (81, 82).

Frailty and deterioration resulting from undernutrition has also been shown in patients with HF (83). Interventions such as the Supplemental Nutrition Assistance Program (SNAP), community partnerships through food pantries, school meals, and community fridges are needed to address food insecurity and health-related comorbidities.

#### Healthcare System

There are well-documented disparities in breast cancer survival and HF by socioeconomic status, access to health insurance, and preventive care. Lack of adequate health insurance leads to high out of pocket medical costs, inability to pay for medications, lack of a primary care physician to perform screening studies, and provide subspeciality referral. The difficulty in navigating screening and treatment for HF or cancer is exacerbated by poverty, lack of insurance, and not having an established continuity clinic. Other socioeconomic factors such as lower education, health literacy, and higher stress levels were associated with lower HF clinic use (84). Patients without health insurance often seek care at safety-net or federally funded hospitals and indigent care clinics. When unable to afford healthcare or medications, patients may need to make trade-offs between basic needs and treatments.

Prevention is a large component in the management of both HF and breast cancer. It has been demonstrated that decreased cancer screening rates are associated with delayed diagnosis and treatment and poorer health outcomes (56). In a study by Kurani and colleagues, 78,302 patients eligible for breast cancer screening living in rural areas were 24% less likely to obtain breast cancer screening than those living in the city. Those living in the most deprived census blocks were 49% less likely to obtain breast cancer screening (85). Interventions such as providing transportation and childcare assistance, providing free screening services, or distributing educational resources through community partnerships have proven to be cost-effective measures at improving quality and length of life by increasing cancer screening (86). As previously outlined, socioeconomic factors effect access to heart failure care and subspeciality clinics (66). Racial disparities also exist in admission for heart failure, referral for diagnostic tests, and administration of advanced heart failure therapies (87, 88).

#### Social Isolation

Finally, the importance of social networks and connections for both breast cancer and HF patients has been well-established. High levels of social support have been shown to be protective for physical and mental health and quality of life (56, 89). In addition, several studies have demonstrated worse all-cause mortality and breast cancer mortality in patients without robust social support (90–92). These studies quantify social support based on both the number of people in the social network as well as the frequency of contact with friends/family following cancer diagnosis. In a study of 2,835 nurses from the Nurses' Health Study, participants that were socially isolated were twice as likely to die as those who were socially connected (90). Those with strong social support were also most likely to adhere to treatment regimens, access healthcare, and treatment options more effectively (93).

One prospective study of HF patients found that 6% of patients experienced severe social isolation; even after controlling for depression, these patients had >3.5 times increased risk of death 68% increased risk of hospitalization, and 57% increased risk of emergency department visits compared to those who did not report social isolation (94). In another study, loneliness was directly associated with more days hospitalized and more readmissions despite equivalent severity of HF (95).

#### Mitigation Strategies

To address the underlying factors that promote both HF and breast cancer, a multi-faceted approach is needed that focuses on SDOH and in turn clinical risk factors (Table 2). A singular theme across all domains of SDOH is a need for a robust income safety net for low-income individuals. Creation of policies that focus SDOH will have a transformational effect on comorbidities that affect HF and breast cancer. Health legislation such as the Patient Protection and Affordable Care Act expanded health insurance, largely through Medicaid, to low-income individuals with cancer and at rates similar to those without cancer (96). This led to increased diagnosis of early-stage breast cancer; however, there was no evidence of increase in timely initiation of cancer treatment due to earlier diagnosis (97). Similarly, although more low-income HF patients were now insured, largely through Medicaid expansion, this did not improve quality of care or in-hospital outcomes in low-income patients with HF (98). These findings underscore a need for an all-encompassing approach, beyond expansion of health insurance, that addresses affordable housing, transportation, food insecurity, access to healthcare, and building social support networks. An intervention such as the Supplemental Nutrition Assistance Program (SNAP) serves as an example for mitigating adverse health outcomes in individuals with food insecurity (99). Working alongside health and social policy makers, community partners, and patients to develop comprehensive intervention strategies that address structural inequities are needed to broaden our view of how to improve health outcomes for breast cancer and HF.

### Reverse Cardio-Oncology

The newer concept whereby HF promotes cancer development is supported by both epidemiological and mechanistic data. In an initial case-control study, HF was associated with nearly 70% higher risk of incident cancer after adjusting for comorbidities (100). This association was present regardless of left ventricular ejection fraction. In a large population-based study of a Danish cohort, individuals with HF had a higher incidence of cancer across different age groups (101). Specifically, there was a 36% higher risk of breast cancer. In addition to incident cancer, two prospective cohort studies in early-stage breast cancer showed a 60% increased risk of recurrence in women who had an interim myocardial infarction (MI) (102). Baseline CVD risk factors, 10-year atherosclerotic CVD risk score, and natriuretic peptide concentrations are associated with increased risk of future cancer (103). Results from observational studies, however, can be biased due to increased surveillance in patients with HF and differences in treatment. Therefore, it is crucial to identify biological pathways that may explain this association.

Animal studies have provided important insights into the association between CVD and cancer. The initial hallmark study evaluated the effect of HF induced by a large anterior MI in mice prone to developing precancerous intestinal tumors (104). Mice with HF had significantly greater tumor growth. Tumor growth was associated with left ventricular dysfunction and myocardial scar. In their panel of candidate proteins, SerpinA3 consistently induced proliferative effects in the tumor via the Akt pathway. There have also been studies specifically evaluating the effect of adverse cardiac remodeling in breast cancer models. In a mouse model of breast cancer, MI induced by coronary artery ligation led to 2-fold increase in tumor growth compared with controls (102). Analysis of the intra-tumoral immune cells showed an increase in monocytic myeloid-derived suppressor cells. These suppressor cells restricted infiltration of anti-tumor cytotoxic T cells, instead promoting pro-tumoral immunosuppressive T regulatory cells. These changes were in part mediated by epigenetic modification of monocytes in the bone marrow.

In a separate breast orthotopic cancer mouse model, pressure overload induced cardiac hypertrophy from transverse aortic constriction led to greater tumor growth and more metastases (105). Tumor growth correlated with the level of cardiac hypertrophy. The authors further identified increased messenger RNA expression of periostin in hypertrophied hearts and increased protein levels in serum. Depletion of periostin from the serum inhibited proliferation of cancer cells while addition of periostin promoted cancer cell proliferation in vitro. Periostin is an extracellular matrix protein that affects cancer cell proliferation, migration, and epithelial to mesenchymal transition. Interestingly, SerpinA3 was not elevated in this mouse model. This may represent differences in early and late stages of cardiac remodeling and HF or mode of cardiac injury. These studies further support the paradigm of reverse cardio-oncology but also reinforce the need for additional studies to better delineate the different pathways that connect CVD to cancer, specifically HF to breast cancer. Greater understanding of the mechanisms would not only allow for targeted therapy but more importantly emphasize the importance of HF and cancer prevention through aggressive risk factor modification by both patients and clinicians.

## Conclusions

**Figure 1:**
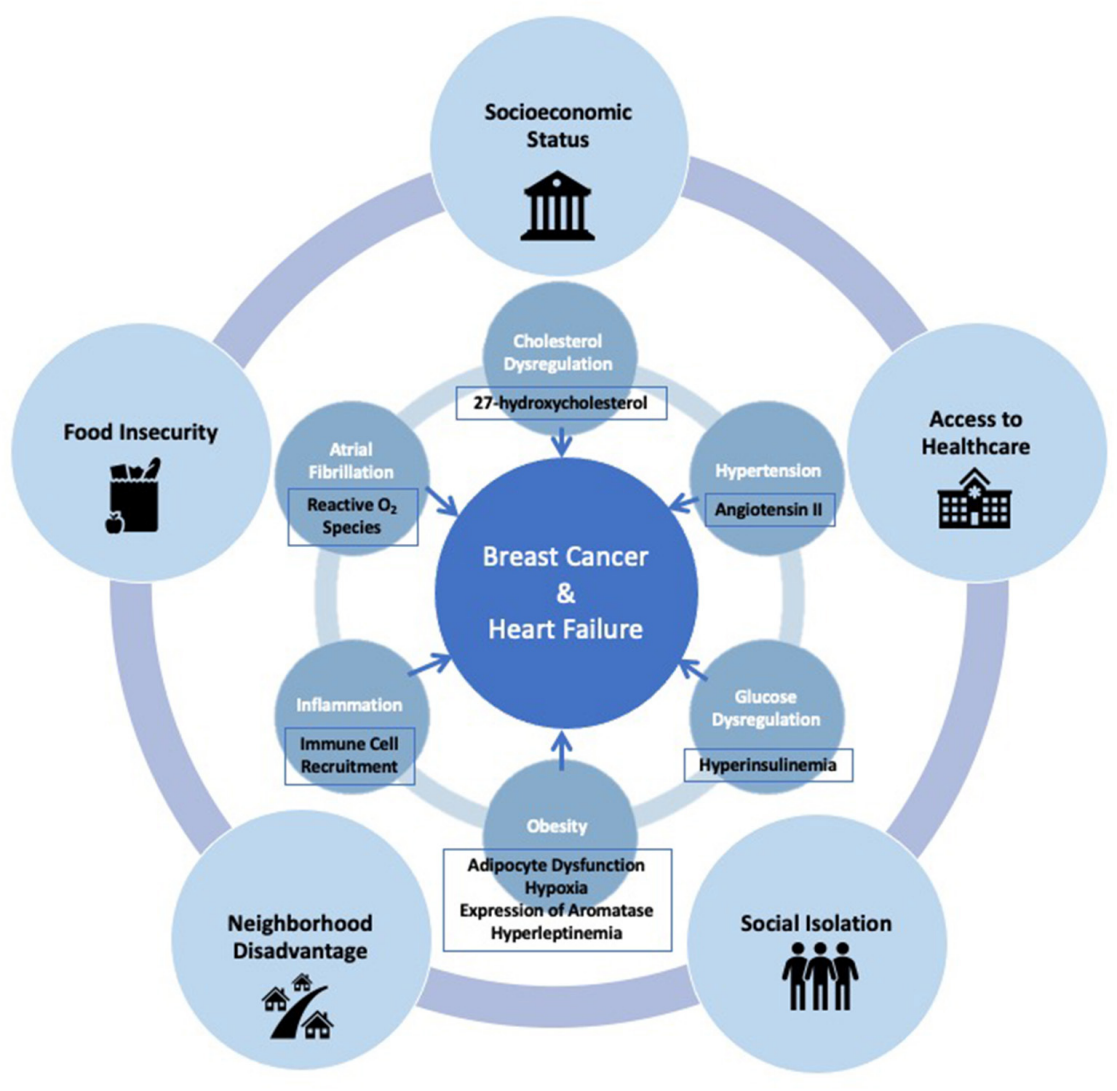
Interconnected social and clinical risk factors, and mechanisms which link the development of breast cancer and heart failure.

The interplay between risk factors associated with breast cancer and HF is very complex. Traditional cardiovascular risk factors, such as obesity, glucose dysregulation, hypertension, cholesterol dysregulation, atrial fibrillation and inflammation, are also closely linked with the development of breast cancer. HF itself has been shown to increase tumor growth and cancer development. Overarching social factors that lead to development of these cardiovascular risk factors, and in turn to breast cancer and HF, must simultaneously be addressed in order to comprehensively develop approaches for prevention of both chronic illnesses (Figure 1). Poverty and inequality are the root causes of several of these social risk factors, such as socioeconomic status, neighborhood disadvantage, food insecurity, an inadequate healthcare system, and social isolation. Implementation of prevention strategies must consider these social factors with equal importance when addressing common risk factors between breast cancer and HF.

## Author Contributions

NA, AS, and AB contributed to concept and design of the review and wrote sections of the the manuscript. NA and AB created the figure. All authors contributed to manuscript revision, read, and approved the submitted version.

## Conflict of Interest

SS has received research grants from the National Institutes of Health (U54 HL160273, R01 HL107577, R01 HL127028, R01 HL140731, and R01 HL149423), Actelion, AstraZeneca, Corvia, Novartis, and Pfizer; and has received personal fees from Abbott, Actelion, AstraZeneca, Amgen, Aria CV, Axon Therapies, Bayer, Boehringer-Ingelheim, Boston Scientific, Bristol-Myers Squibb, Cardiora, Coridea, CVRx, Cyclerion, Cytokinetics, Edwards Lifesciences, Eidos, Eisai, Imara, Impulse Dynamics, Intellia, Ionis, Ironwood, Lilly, Merck, MyoKardia, Novartis, Novo Nordisk, Pfizer, Prothena, Regeneron, Rivus, Sanofi, Shifamed, Tenax, Tenaya, and United Therapeutics. The remaining authors declare that the research was conducted in the absence of any commercial or financial relationships that could be construed as a potential conflict of interest.

## Publisher's Note

All claims expressed in this article are solely those of the authors and do not necessarily represent those of their affiliated organizations, or those of the publisher, the editors and the reviewers. Any product that may be evaluated in this article, or claim that may be made by its manufacturer, is not guaranteed or endorsed by the publisher.
